# Current Challenges in Plant Eco-Metabolomics

**DOI:** 10.3390/ijms19051385

**Published:** 2018-05-06

**Authors:** Kristian Peters, Anja Worrich, Alexander Weinhold, Oliver Alka, Gerd Balcke, Claudia Birkemeyer, Helge Bruelheide, Onno W. Calf, Sophie Dietz, Kai Dührkop, Emmanuel Gaquerel, Uwe Heinig, Marlen Kücklich, Mirka Macel, Caroline Müller, Yvonne Poeschl, Georg Pohnert, Christian Ristok, Victor Manuel Rodríguez, Christoph Ruttkies, Meredith Schuman, Rabea Schweiger, Nir Shahaf, Christoph Steinbeck, Maria Tortosa, Hendrik Treutler, Nico Ueberschaar, Pablo Velasco, Brigitte M. Weiß, Anja Widdig, Steffen Neumann, Nicole M. van Dam

**Affiliations:** 1 Leibniz Institute of Plant Biochemistry, Stress and Developmental Biology, Weinberg 3, 06120 Halle (Saale), Germany; Sophie.Nahrstedt@ipb-halle.de (S.D.); Christoph.Ruttkies@ipb-halle.de (C.R.); Hendrik.Treutler@ipb-halle.de (H.T.); sneumann@ipb-halle.de (S.N.); 2 German Centre for Integrative Biodiversity Research (iDiv) Halle-Jena-Leipzig, Deutscher Platz 5e, 04103 Leipzig, Germany; anja.worrich@idiv.de (An.W.); alexander.weinhold@idiv.de (Al.W.); helge.bruelheide@botanik.uni-halle.de (H.B.); yvonne.poeschl@idiv.de (Y.P.); christian.ristok@idiv.de (C.R.); anja.widdig@eva.mpg.de (A.W.); nicole.vandam@idiv.de (N.M.v.D.); 3 Institute of Biodiversity, Friedrich Schiller University Jena, Dornburger-Str. 159, 07743 Jena, Germany; 4 UFZ—Helmholtz-Centre for Environmental Research, Department Environmental Microbiology, Permoserstraße 15, 04318 Leipzig, Germany; 5 Applied Bioinformatics Group, Center for Bioinformatics, University of Tübingen, Sand 14, 72076 Tübingen, Germany; alka@informatik.uni-tuebingen.de; 6 Leibniz Institute of Plant Biochemistry, Cell and Metabolic Biology, Weinberg 3, 06120 Halle (Saale), Germany; Gerd.Balcke@ipb-halle.de; 7 Institute of Analytical Chemistry, University of Leipzig, Linnéstr. 3, 04103 Leipzig, Germany; birkemeyer@chemie.uni-leipzig.de; 8 Institute of Biology/Geobotany and Botanical Garden, Martin Luther University Halle-Wittenberg, Am Kirchtor 1, 06108 Halle (Saale), Germany; 9 Molecular Interaction Ecology, Institute for Water and Wetland Research (IWWR), Radboud University, Heyendaalseweg 135, 6525 AJ Nijmegen, The Netherlands; owcalf@gmail.com (O.W.C.); mirkamacel@gmail.com (M.M.); 10 Department of Bioinformatics, Friedrich Schiller University Jena, Ernst-Abbe-Platz 2, 07743 Jena, Germany; kai.duehrkop@uni-jena.de; 11 Centre for Organismal Studies, Heidelberg University, Im Neuenheimer Feld 360, 69120 Heidelberg, Germany; emmanuel.gaquerel@cos.uni-heidelberg.de; 12 Weizmann Institute of Science, Faculty of Biochemistry, Department of Plant Sciences, 234 Herzl St., P.O. Box 26, Rehovot 7610001, Israel; Uwe.Heinig@weizmann.ac.il (U.H.); nirshachaf@gmail.com (N.S.); 13 Institute of Biology, University of Leipzig, Talstraße 33, 04109 Leipzig, Germany; marlen-k@gmx.net (M.K.); brigitte_schloegl@eva.mpg.de (B.M.W.); 14 Chemical Ecology, Bielefeld University, Universitätsstr. 25, 33615 Bielefeld, Germany; caroline.mueller@uni-bielefeld.de (C.M.); rabea.schweiger@uni-bielefeld.de (R.S.); 15 Institute of Informatics, Martin Luther University Halle-Wittenberg, Von-Seckendorff-Platz 1, 06120 Halle (Saale), Germany; 16 Institute of Inorganic and Analytical Chemistry, Friedrich Schiller University Jena, Lessingstr. 8, 07743 Jena, Germany; Georg.Pohnert@uni-jena.de (G.P.); christoph.steinbeck@uni-jena.de (C.S.); Nico.Ueberschaar@uni-jena.de (N.U.); 17 Group of Genetics, Breeding and Biochemistry of Brassica, Misión Biológica de Galicia (CSIC), Apartado 28, 36080 Pontevedra, Spain; vmrodriguez@mbg.csic.es (V.M.R.); mtortosa@mbg.csic.es (M.T.); pvelasco@mbg.csic.es (P.V.); 18 Department of Molecular Ecology, Max Planck Institute for Chemical Ecology, Hans-Knöll-Straße 8, 07745 Jena, Germany; mschuman@ice.mpg.de; 19 Research Group of Primate Kin Selection, Max Planck Institute for Evolutionary Anthropology, Deutscher Platz 6, 04103 Leipzig, Germany

**Keywords:** biochemistry, bioinformatics, ecology, ecometabolomics, metabolites

## Abstract

The relatively new research discipline of Eco-Metabolomics is the application of metabolomics techniques to ecology with the aim to characterise biochemical interactions of organisms across different spatial and temporal scales. Metabolomics is an untargeted biochemical approach to measure many thousands of metabolites in different species, including plants and animals. Changes in metabolite concentrations can provide mechanistic evidence for biochemical processes that are relevant at ecological scales. These include physiological, phenotypic and morphological responses of plants and communities to environmental changes and also interactions with other organisms. Traditionally, research in biochemistry and ecology comes from two different directions and is performed at distinct spatiotemporal scales. Biochemical studies most often focus on intrinsic processes in individuals at physiological and cellular scales. Generally, they take a bottom-up approach scaling up cellular processes from spatiotemporally fine to coarser scales. Ecological studies usually focus on extrinsic processes acting upon organisms at population and community scales and typically study top-down and bottom-up processes in combination. Eco-Metabolomics is a transdisciplinary research discipline that links biochemistry and ecology and connects the distinct spatiotemporal scales. In this review, we focus on approaches to study chemical and biochemical interactions of plants at various ecological levels, mainly plant–organismal interactions, and discuss related examples from other domains. We present recent developments and highlight advancements in Eco-Metabolomics over the last decade from various angles. We further address the five key challenges: (1) complex experimental designs and large variation of metabolite profiles; (2) feature extraction; (3) metabolite identification; (4) statistical analyses; and (5) bioinformatics software tools and workflows. The presented solutions to these challenges will advance connecting the distinct spatiotemporal scales and bridging biochemistry and ecology.

## 1. Introduction

Technological advances in chromatography coupled with mass spectrometry permit snapshots of nearly all low molecular weight (typically 50–1000 Da) polar and semi-polar metabolites in organisms at once, without targeting specific biochemical compounds [1]. This technology is called “metabolomics” and is now used widely in biochemistry and biotechnology for various types of organisms, including plants, soil microbiota and mammals [2,3,4]. There are several metabolomics acquisition techniques, but liquid chromatography coupled with mass spectrometry (LC/MS), gas chromatography coupled with MS (GC/MS) and nuclear magnetic resonance spectroscopy (NMR) are the most commonly used methods (for explanation of the techniques, see [5,6,7]; Table 1 and Table 2).

Metabolites are key components in both biochemical and ecological processes. To survive and successfully reproduce in their natural habitats, organisms need to adjust their morphological and physiological characteristics in response to varying environmental conditions, as well as to interactions with other organisms [8]. These ecophysiological adjustments can be identified and quantified using metabolomics techniques [9,10,11,12,13]. The great advantage of metabolomics is that it can be applied to any species without prior knowledge of its biochemical or genetic composition. This universality and the coverage of a wide range of bioactive compounds initiated a new research field called “Eco-Metabolomics”—the application of metabolomics to ecology and, thus, understanding the biochemical mechanisms governing species interactions with the environment and with other organisms [8,10,11,12,14].

It is estimated that there are between 200,000 and 1,000,000 metabolites in the plant kingdom, of which about 51,000 (as listed in the KNApSAcK database 2018-02-14, [15]) have been found in higher plants [15,16]. However, many of the known compounds have been identified only in model organisms. Although metabolomics is one of many tools available in chemical ecology, its wide compound coverage sets it apart from “classic” approaches. Metabolomics allows for new strategies to discover novel compounds and their functioning in ecosystems particularly when including non-model species [17,18].

Thus, there is a growing interest to apply Eco-Metabolomics to various levels of biodiversity research, ranging from individuals, populations and communities to whole ecosystems. Metabolomics allows the analysis of chemical variation among non-model organisms with regard to one or more ecological factors. Moreover, it may result in the discovery of metabolomic traits that explain ecosystem functioning or community assembly [2,19,20,21]. Eco-Metabolomics approaches promise to reveal the biochemical basis of various ecological interactions. In ecology and biodiversity research, organisms are often sampled from natural or semi-natural environments and, as a result, many large field experiments have been set up. For example, biodiversity ecosystem functioning (BEF) experiments such as Cedar Creek, BIODEPTH, the Jena Experiment or BEF-China comprise plant communities, varying in plant species richness [22,23,24,25]. They are originally designed to investigate the relationship between plant diversity and a wide range of ecosystem functions, but also address the effects of environmental factors such as soil type, temperature, fertilization, disturbances and interacting organisms. The worldwide CTFS-ForestGEO network has been established to understand the impact of climate change on forest ecosystems [26]. With such large experimental facilities, basic ecological growth and performance parameters, as well as physiological responses of plants to ecological factors are measured over long time periods [27]. In addition, national and international networks and programmes such as the US NSF National Ecological Observatory Network (NEON), the Nutrient Network (NutNet) or the Long Term Ecological Research (LTER) sites have been set up to promote such research activities worldwide [28,29,30]. Even though metabolomics analyses have not yet been included in most of these and other ecological research projects, there is a huge potential to apply metabolomics in field experiments with designed and controlled complexity.

Typically, these field experiments are accompanied by collections of large data sets that require advanced biostatistical analyses. When metabolomics analyses are applied, the magnitude of the data collection will increase considerably. In mass spectrometry (MS), data are comprised of thousands of chemical features that are described by retention time (RT) and mass-to-charge-ratio (*m*/*z*) [31]. Moreover, metabolite matrices are merely starting points for sample classification and further structural identification [1,32]. To identify the ecological function of metabolic shifts, further data are usually included in the form of species-related traits and environmental variables. In this context, there is an urgent need for sophisticated bioinformatics tools that help to characterize metabolic shifts in organisms in response to various ecological interactions [33].

In this review, we focus on plants and their biochemical interactions at various ecological levels. These include trophic and other interaction networks such as plant–plant, plant–herbivore, plant–pathogen, plant–environment and plant–soil. We include examples of where metabolomics has been applied to diversity research and also discuss related examples from other domains. First, we analyse how ecology and biochemistry traditionally approach research from two different directions. Then, we explore the importance of Eco-Metabolomics linking biochemistry and ecology. Finally, we discuss current challenges and present recommendations.

## 2. What Is Eco-Metabolomics?

While there are many different definitions and views on Eco-Metabolomics, it can be understood as the application of metabolomics techniques in ecological studies to characterise biochemical mechanisms underlying interactions of organisms with the environment and with other organisms across different spatial and temporal scales. Metabolomics either characterises metabolites in a sample following an untargeted approach without necessarily identifying metabolites (metabolic fingerprinting) or uses semi-targeted approaches that focus on specific groups of metabolites or specific pathways (metabolite profiling) [34]. Eco-Metabolomics employs both approaches to provide biochemical evidence for ecological processes, e.g., plant growth, phenotypic responses, morphological adaptations to environmental changes or responses to other organisms such as pathogens, herbivores, competitors, parasites or symbiotic organisms at coarser scales of spatiotemporal complexity. The main distinctions between Eco-Metabolomics and chemical ecology are the complex experiment designs, especially with field experiments focusing on species interactions in communities and ecosystems and the acquisition and concomitant analysis of a multitude of metabolites in a singular approach [17]. When compared to typical metabolomics, these characteristics and the use of non-model species reduce the numbers of “true replicates” and cause additional random variation created by the variability in genetic background and the natural environment.

## 3. Current Research

**Figure 1:**
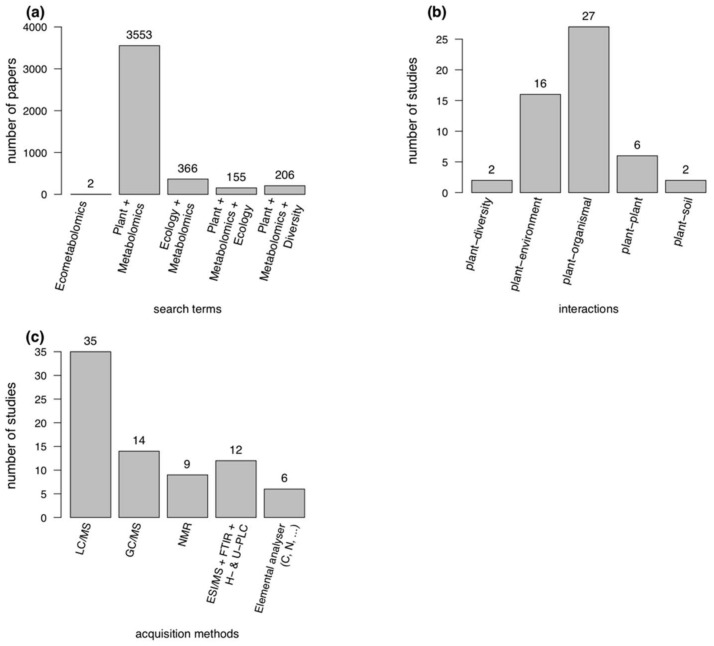
Search hits for terms related to Eco-Metabolomics in PubMed in the last decade: (**a**) search hits by specific terms; (**b**) number of original research studies in Table 1 targeting a specific interaction level; and (**c**) number of original research studies in Table 1 that used specific metabolomics acquisition methods.

The term “Eco-Metabolomics” (or “Ecometabolomics”) is not yet well established in the scientific community. However, there is a growing number of publications that use cognate terms either in the abstract or as part of the keywords (Figure 1). To find studies related to “Eco-Metabolomics”, search terms such as “metabolomics + ecology” or “metabolomics + diversity” were used in public databases such as PubMed (Figure 1a). Table 1 and Table 2 show an overview of some selected research papers in the discipline of Eco-Metabolomics.

We found 53 Eco-Metabolomics studies that performed experiments at various interaction levels (Figure 1b and Table 1). In total, 57% of these studies were performed with cultures or in chambers or greenhouses and 45% followed a bottom-up approach (Table 1, see Section 4). Overall, 43% performed field experiments and 55% realized a top-down approach (Table 1, see Section 4). Especially these latter studies used non-model species as study subjects. Most studies (85%) identified compounds or compound classes or had an acquisition method that targeted specific compound classes (Table 1). The most common metabolomics acquisition methods were LC/MS, GC/MS and NMR (Figure 1c). Some studies used additional methods such as (U)HPLC without MS and elemental analysers to assist metabolomics (Figure 1c).

## 4. Bridging the Gap between Biochemistry and Ecology

**Figure 2:**
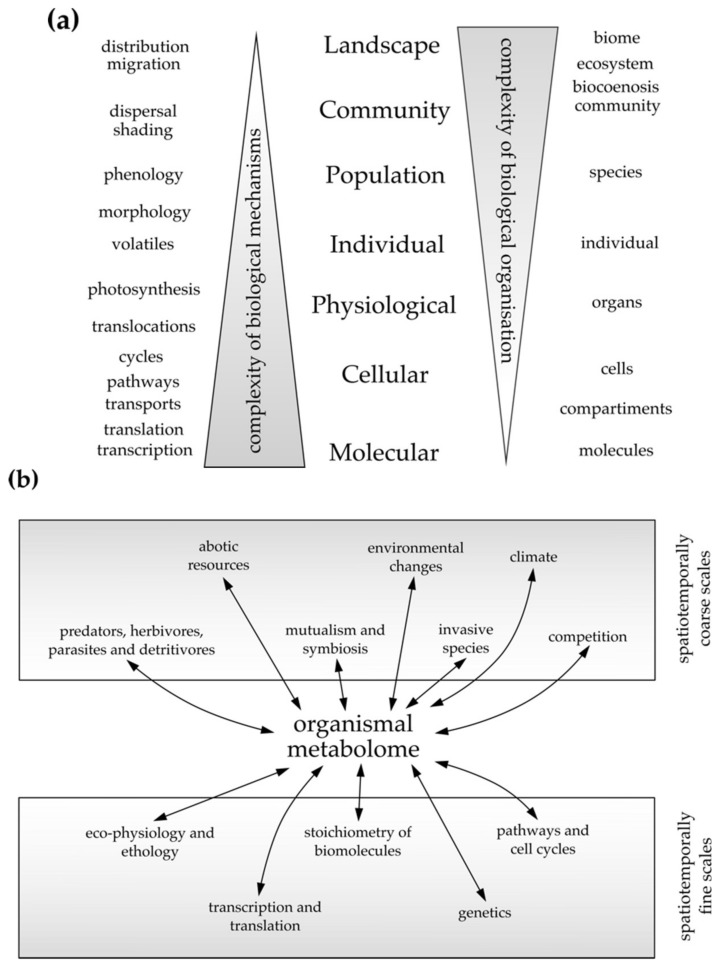
Spatiotemporal scales and the central position of Eco-Metabolomics as a mediator between biochemical and ecological scales. (**a**) Spatiotemporal scales and levels of complexity. The different spatiotemporal scales are listed in the centre. Exemplary mechanistic processes and their association with particular spatiotemporal scales are listed on the left. Exemplary organisational entities and their association with spatiotemporal scales are listed on the right. (**b**) Central position of the organism metabolome and some interactions acting at different spatiotemporal scales. Figures modified after references [14,107].

Traditionally, the fields of biochemistry and ecology operate at distinct spatiotemporal scales with different biochemical resolution (Figure 2a). For example, initially, biochemistry and chemical ecology explored the diversity of natural products with the goal to identify the specific compounds that underlie isolated organismal interactions. This view has been challenged as many compounds have been identified to be multifunctional across spatial and temporal scales and appear to be also involved in multiple organismal interactions [17]. In contrast, Eco-Metabolomics is an integrative multidisciplinary research discipline that has emerged to conciliate these different scales.

As illustrated by [92], climate change has impacts on multiple scales. Altered temperature and moisture conditions can modify gene expression and the biochemistry of plants, which act at different scales within the species (Figure 2a). At these intrinsic scales, biochemical responses can be detected by measuring changes in metabolite levels [95]. At the same time, the different individual responses of plant species in the community modify species composition acting at population and community scales [110,111]. An outcome can be species migration, which is apparent at spatiotemporally coarse scales ranging from a few up to several hundred kilometres (Figure 2a). Finally, all these different kinds of changes affect ecosystem services and thus indirectly human well-being [107]. In each of these processes, metabolite profiles can be used to measure plant responses with different biochemical resolution [112]. They can also be used to identify biochemical traits that can serve as marker, e.g., for phenotypic plasticity, chemical interactions with other organisms or resolving the invasive potential of exotic plants [37,47,110]. Thus, Eco-Metabolomics is a discipline which allows researchers to describe interactions between processes acting at different spatiotemporal scales. Because it uses metabolites for mechanistically describing these processes, it can be seen as the mediator between different research approaches [94]. Bioinformatics and biostatistical tools are important during the entire data processing and data analysis pipeline [46].

In the following, two different approaches are presented. The “bottom-up” approach is typically taken by biochemists who infer from spatiotemporally fine scales within plants (e.g., processes in cells, physiological traits, growth) to spatiotemporally coarser scales (e.g., population fitness, biomass, yield of crops) (Figure 2a,b). By contrast, the “top-down” approach is typically taken by ecological studies that infer from spatiotemporally coarse scales (e.g., interaction processes at population and community scales) to spatiotemporally finer scales (e.g., identifying morphological, physiological and biochemical traits of plants). Table 1 lists studies and orders them according to the approach taken.

### 4.1. The Bottom-Up Approach, Inferring from Cellular to Individual Spatiotemporal Scales

Traditionally, biochemistry mainly targets intrinsic processes in individual organisms. For example, the role of biochemical compounds is elucidated in biological pathways acting at the cellular scale or physiological processes on the scales of organs of well-known model species (Figure 2a). In Eco-Metabolomics, both systemic and intrinsic physiological responses to environmental factors are studied in model and non-model species (Figure 2a,b). To understand the relevance of these biochemical responses it is pivotal to identify metabolites that are modulated under certain conditions or that distinguish individuals or species interacting at population and community scales [6,112]. Such metabolites can describe processes at spatiotemporally coarser scales such as changes in yield and biomass of crops, or pinpoint changes in species interactions at population and community scales.

For example, [72] investigated how the metabolome of tomato fruits changes with different salinity levels and observed carotenoid accumulation with higher salinity. [71] found plastic responses of leaves of different maize lines to different temperature conditions and identified metabolites associated with heat and cold stress. Similarly, foliar metabolic changes related to drought stress were studied in *Arabidopsis thaliana* [13]. Symbiotic interactions between several plant species and an arbuscular mycorrhizal fungi (AMF) have been studied by [74]. They annotated foliar metabolites that are shared between species, those that are species-specific as well as overlapping leaf metabolic responses to AMF. [84] described allelochemicals in tobacco that are produced in leafy galls induced by a fungal pathogen. Moreover, [82] analysed plant metabolome changes in response to nematode and aphid interferences in roots and shoots and found that the responses highly depend on the fertilization status of the plant [83].

The examples above demonstrate that a bottom-up research approach is common in biochemistry (Figure 2a,b). Here, metabolites are studied and conclusions are drawn from intrinsic processes (e.g., genes, metabolites and pathways). These are then related to higher levels of organisation, usually from plant cells or organs to individuals or from individuals up to plant populations. At fine scales, the complexity of biological mechanisms is large and turn-over of processes such as translation into molecules, enzymatic activity, biochemical pathways and cell cycles occur within seconds to a few hours [107].

To control for this complexity, biochemical research is typically carried out with model species (e.g., *A. thaliana*, *Medicago*, tobacco or tomato). The increasing knowledge of the role of metabolites in these model species also allows the analysis of non-model species which are more commonly used in Eco-Metabolomics. This often goes along with more complex experimental designs. For example, [80] realized a three-factorial approach. They studied the responses of *Brassica oleracea* to leaf age, herbivory and drought stress. Similarly, [83] studied interference of two herbivores (one aphid and one nematode species) and two fertilization conditions simultaneously. [68] performed a glasshouse experiment with two *Echium* species and identified root shikonins at the physiological level to play an important role with plant phenological stage at the population scale (refer also Table 1 for more examples).

Many of the above studies used untargeted approaches to determine the different states of organisms. However, metabolomics techniques can also be efficiently used for the identification of true mediators of interactions [102]. If there is already some knowledge of the chemical properties, targeted profiling can be used to identify candidate metabolites. Their function can be confirmed by bioassays as e.g., demonstrated by the identification of the first sex-pheromone of unicellular diatoms [113].

### 4.2. The Top-Down Approach, Inferring from Coarse to Fine Spatiotemporal Scales

By contrast, in ecological experiments, environmental effects and biotic interactions of organisms are studied. This is achieved at spatiotemporally coarse scales, e.g., at the population scale (in which intraspecific differences are mainly studied) or at the community scale (where responses of different plant species in an ecosystem are studied) [110]. Processes at these scales occur over a time-span of hours (e.g., along with diurnal cycles) up to several years (e.g., species migration and community changes as a response to climate change). At these coarse scales, complexity of interactions between organisms is expected to be larger than intrinsic biological mechanisms (Figure 2b) [107].

Only a few studies analysed metabolites at the community scale, which is probably due to the complexity and the large number of profiles necessary to be acquired. For example, in an analysis of community assembly, [50] found that 37 species of *Inga* trees share herbivores and pathogens at local and regional spatial scales. Their results showed that these interactions are also important for niche differentiation of different congeneric *Inga* species in the community. [56] found that biochemically diverse assemblages facilitate ecological coexistence and that interspecific variation permits niche segregation among congeneric tree species based on chemical defences. Other studies reported that metabolite profiles depend on the diversity level, strength of competition and neighbouring plants [35,36,60] (Table 1). In a case study with soil bacterial communities, [114] showed that there are metabolic relationships between soil species richness, niche breadth and distribution. It is increasingly acknowledged that the rhizosphere comprises a highly diverse community of micro-organisms which interact with root exudates [93]. We expect that comparable studies analysing rhizosphere metabolomes interacting with plants will yield similarly novel insights and may use comparable methodological approaches as with, e.g., community metabolomics of microbe colonies [115].

At the population scale, typically several individuals of one species are studied with regard to environmental or organismal changes. [38] showed in a field experiment with wild *Carex caryophyllea* that differences in foliar metabolite profiles can be linked to genetic diversity, edaphic conditions and growth-related traits. [64] revealed that the shoots and roots of the two grasses *Holcus lanatus* and *Alopecurus pratensis* responded differently to drought and warming. In a different experiment with the shrub *Sambucus nigra*, these authors showed that there are specific interactions between plants and the microbial community in the phyllosphere [65]. In a greenhouse experiment, [40] found that exotic species have more, and also more unique, metabolites when compared to native congeners. They experimentally assessed that a generalist herbivore species performs worse on exotics. Thus, the authors provided evidence for a hypothesis in invasion biology—the “Novel weapons hypothesis” [116].

In contrast to biochemistry, in ecology, a top-down research approach is common (Figure 2a). Complex ecological processes are broken down into smaller sets and studied individually to reveal cryptic biochemical traits that act upon the different species [89]. In this context, highly specific metabolites (sometimes also referred to as biomarkers) may serve as proxies for “eco-chemical” traits and allow for a functional classification based on metabolites [117]. Whereas in biochemistry compounds play a pivotal role in identifying mechanistic components of processes, in ecology this role is traditionally fulfilled by traits, e.g., morphological, physiological or phenological characteristics of individual plants. Some Eco-Metabolomics studies found biochemical traits that describe, for example, relationships with plant phenological stage [68], foliar chemical defences of trees [46,54] or plant defence traits in native and non-native populations [49,76]. More examples on how to use Eco-Metabolomics to find “eco-chemical” traits that describe ecological processes such as species coexistence, (multi-)trophic interactions and phenotypic plasticity can be found in the reviews of [90,91,112] (Table 2).

## 5. Current Challenges

To identify the specific compounds which have an impact in ecology, it is necessary to “scale up” from biochemical to ecological scales. For example, some insects are capable of smelling volatiles emitted by flowers over long distances [118], and fruit bats as well as frugivorous primates use fruit odours to detect ripe fruits [119,120,121]. Thus, the production of volatiles in plants can have great impact on pollination [122,123] and dispersal [124,125]. To identify volatiles, metabolomics techniques such as GC/MS can be used. However, within the plethora of substances that are produced during plant–animal interactions, it is still a challenge to pinpoint the compounds, or combinations thereof, which employ key ecological functions [118,122,126,127].

When compared to other analytical techniques, NMR has the advantage of covering both polar and non-polar metabolites and allows their identification by comparing resonance frequencies and line shapes in the spectra wit spectral libraries [20,128]. Data processing is very complex as raw data have to be pre-processed, e.g., into so-called bucketing tables. Due to the numerous approaches to generate these bucketing tables [129,130] and the high number of different tools and approaches available for these processing steps [131,132], NMR data processing and metabolite identification remain very challenging.

Similarly, in Eco-Metabolomics, there is also the challenge to “scale down” from ecological to biochemical scales, i.e., to find important (sets of) metabolites in different organisms (“eco-chemical traits”, see above) that can be linked to particular ecological interactions. As organisms in ecosystems produce a multitude of different metabolites, appropriate experimental designs and biostatistical methods are necessary to select the candidates that e.g., underlie diversity [35] or can be attributed to specific interactions [52,80].

Eco-Metabolomics can also be applied to both sides of the organismal interaction, for example between plants and herbivores. By defining the concept of the “metabolic interface” between plants and caterpillars, [133] could identify coumaroylquinic acids. This group of bioactive compounds was enriched in both jasmonic-acid induced plants and caterpillars feeding on them. By going up the trophic chain, they are likely to affect higher trophic organisms, e.g., the larvae of endoparasitic wasps [134], which feed on the caterpillar’s fatbody and have no direct contact to the plant.

For top-down approaches in Eco-Metabolomics, identification of single metabolites is often not feasible. Here, a global analysis of samples such as metabolic fingerprint analysis is performed in which sets of features are analysed instead of singular features [34,135,136]. Generally, metabolic fingerprints are generated for metabolites that are shared between or which are distinct for the different species [137,138].

In a three-day workshop held at the German Centre for Integrative Biodiversity Research (iDiv) Halle-Jena-Leipzig from 16 to 18 October 2017, the authors collected current challenges in Eco-Metabolomics (Table 1). In a collaborative effort, the participants identified five key challenges that many Eco-Metabolomics studies had to solve prior to making conclusion. These challenges are presented as follows.

### 5.1. Complex Experimental Designs and Large Variation of Metabolite Profiles

Experiments with model species such as *A. thaliana* are usually carried out with known genotypes under well-controlled conditions in green houses and growth chambers (Table 1). By contrast, typical Eco-Metabolomics studies often require more complex experimental designs as they are often carried out under field conditions and with non-model species [6,105] (Table 1). As a result, metabolite profiles obtained in many Eco-Metabolomics experiments are further modulated by different genetic backgrounds and life-stages of the individuals, by short- and long-term environmental fluctuations, such as season and weather patterns, and also by varying histories of biotic interactions. Thus, the variability among metabolite profiles within treatment groups is usually much larger than in conventional metabolomics studies (Table 1).

To assess the level of variation within treatment groups (groups of replicates), large-scale experiments may require conducting a pilot study. For Eco-Metabolomics, this is still uncommon (Table 1). However, pilot studies allow for an estimation of the number of samples needed (and thus, number of necessary metabolite profiles to be acquired) to verify effect sizes in a statistically clean way [139]. There are freely available R packages (pwr or MBESS) and templates to support scientists with the corresponding statistics [140].

Many ecological experiments that target population and community scales are designed in such a way that the highest number of replications are created at the level where the largest variability among samples is expected [139,141,142]. As variability often increases with spatial or temporal scales, sampling campaigns with a multi-level block design and randomized positioning of samples in the blocks are realised [141]. Eco-Metabolomics experiments realising a top-down approach and covering spatiotemporally coarse scales are usually designed similarly complex (e.g., [56]; Table 1). With bottom-up approaches, which are often carried out in glasshouses or growth chambers, establishing the appropriate number of controls is also vital in order to ensure effect sizes between control and treatments (Table 1).

Metabolite profiles are generally acquired under as stable conditions as possible. The sampling is typically performed within a short time interval at a defined time of the day to avoid fluctuations due to e.g., circadian rhythms. Similar weather conditions are preferred, i.e., with sunlight and no rainfall prior to and during sampling as they are known to influence nutrient uptake and concentrations of metabolites [143,144,145]. Just as with conventional metabolomics studies, any metabolic activity in the samples needs to be inactivated as rapidly and efficiently as possible. If samples are collected without access to liquid nitrogen and immediate sample storage at −80 °C, as is often the case with ecological field studies, alternative protocols for sampling and storage are used, e.g., use of dry ice in field boxes [146].

Many top-down approaches that operate at spatiotemporally coarse scales aim at masking the complexity of intrinsic biological mechanisms (Figure 2a) by analysing mechanisms of biological organisation which are mediated by sets of “eco-chemicals” [36,45,89]. The processing of samples and the general metabolite acquisition strategy is often very specific to the underlying research question (Table 1). Large sampling campaigns at community and population scales often require samples to be pooled and homogenised (e.g., leaves from one plant individual are pooled into one sample) [142,147]—a strategy that is not (yet) followed by many studies (Table 1). Using pooled samples improves the reproducibility of measurements by diminishing spatial heterogeneities, but sometimes impedes detailed insights at spatiotemporally fine scales. This necessitates different experimental designs or methodologies (see below).

More specific interactions, such as those between plants and herbivores or pathogens, are usually analysed at population or individual scales. Here, it is important to increase the spatial resolution and obtain detailed insights on intrinsic biological mechanisms in both plants and associated organisms (Table 1). Studies that pursue a bottom-up approach restrict the sampling of plant material to the specific organs that are affected by the plant–organism interaction (e.g., plant leaves, mycorrhizal roots) [40,51,75,76]. Furthermore, considering alternative approaches such as MALDI-TOF-MS or fluorescence imaging, which are not covered in this review, can provide detailed insights into (sub-)cellular localization of specific metabolites that underlie plant–enemy interactions [95,148,149].

There are *m/z* or RT shifts within the subject samples due to matrix effects and within the instrument run due to batch effects. Quality control (QC) of the analytical setup and interspersal of QC and mixed-QC samples is necessary to detect and correct these shifts—a strategy which has not yet been implemented in many Eco-Metabolomics studies [147] (Table 1). To correct for shifts, a regression can be performed between peaks in each sample [150]. In general, instrumental configuration and the type of separation technique influence the analytical reproducibility of metabolomics experiments for both MS and NMR platforms [128,151,152]. As with conventional metabolomics experiments, analytical normalization strategies including a reasonable number of blanks are to be employed to separate batch-to-batch effects in instrumental analysis and variances during sampling [153,154].

### 5.2. Feature Extraction

From the raw metabolite profiles, metabolite features need to be extracted using bioinformatics tools. In this review, we focus on XCMS and OpenMS (see [155,156]) even though we found many studies that used other tools and algorithms that are available to process and align raw metabolite data (Table 1 and Table 3; [132,157]). Bioinformatical operations on the raw data, such as peak detection, feature extraction, feature alignment and retention time shift correction were initially designed for data generated on model organisms. For Eco-Metabolomics data, feature extraction and alignment need to be optimized to deal with different organisms, multi-factorial experiment designs and the resulting large variability of samples.

Mass spectrometry raw data are usually processed by optimizing parameter settings such as signal-to-noise thresholds and maximal *m*/*z* deviations for peak detection for the particular analytical setup. Entering optimized parameters will help peak detection algorithms to separate peaks from the noise reliably, align corresponding features across samples in a correct manner and assign unique feature identifiers [155]. In addition, performing mathematical transformations (e.g., log or sqrt) on the feature matrix may be necessary to reach a semi-normal distribution of values as far as possible [139,158]. In OpenMS, the tool TOPPView can be used to guide the manual parameter optimization by visualizing the results of the feature detection step (“FeatureFinderMetabo”) [159]. Different layers, corresponding to features extracted using different parameter settings, can be compared to separate features reliably [160].

However, when compared to conventional metabolomics experiments, Eco-Metabolomics experiments with complex designs may require different parameter settings for feature alignment—called “grouping” in XCMS or “feature linking” in OpenMS—to correctly match extracted features between different samples [31,161]. Although parameter selection depends on experimental design, level of variability and the type of analytical platform used, many Eco-Metabolomics studies that used XCMS applied the following settings appropriate for LC/MS profiles and the centWave algorithm (Table 1). The parameter “minfrac” specifies for each feature the minimum fraction of occurrence in a class (e.g., treatment group) to be valid was chosen between 0.3 and 0.6 to address the large variability between the different kinds of samples. Furthermore, parameter values for “ppm” (describes the maximum tolerated *m*/*z* deviation) were often chosen between 5 and 30 of (parameter “ppm” in XCMS), values for “bw” (bandwidth, accounting for slight retention time deviations, for grouping features) between 3 and 5, and values for “snthresh” (signal-to-noise cut-off) between 2 and 5. For GC/MS, larger values for “ppm” and “bw” may be required for Eco-Metabolomics applications.

For certain experimental designs, it could be reasonable to perform the grouping step in XCMS for each block separately and to merge the resulting peak tables afterwards. Block here refers to the arrangement of experimental units in a statistical test. According to the guidelines on good scientific practices, one should never perform grouping according to the treatment groups among the differences in metabolite composition are tested. Integrating areas of missing peaks between samples (e.g., using the method “fillPeaks” in XCMS) is expected to not work reliably due to the large variability between different groups of samples (species, treatments, sampling times). In OpenMS, linking is rather flexible and can be adjusted to fit the experimental design [156]. Multiple linking steps can be performed consecutively. For example, all samples from two groups or treatments can be linked separately followed by linking both groups to obtain their consensus features. Using TOPPView, these can again be visualized and evaluated using different parameter settings for RT and *m/z* distances [160].

LC/MS and GC/MS feature matrices usually include redundant information in the form of adducts, isotopes and in-source fragments. These are important for metabolite identification (see below), but can also lead to collinearity of features (linear relationships of features or fragments that belong to the same feature in the peak table). Collinearity may be a problem with some subsequent statistical analyses. For instance, in the R package CAMERA [162], collinearity can be reduced by aggregating features that were categorized by CAMERA into the same pseudo compound group. [163] proposed the function “getReducedPeaklist” to CAMERA (version 1.33.3 or later) that can be used instead of the regular “getPeaklist” function.

### 5.3. Metabolite Identification

With many acquisition methods, the identification of features is still a challenge [7]. In addition, in Eco-Metabolomics, non-model organisms are used that have a high number of truly novel compounds, called “unknown unknowns” [164]. The gold standard for compound identification is the comparison of the obtained MS data with that of a reference standard. However, for the novel compounds detected in the non-model species, there is a dire lack of reference standards. Moreover, it may be challenging to purify sufficient amounts of unknown compounds to sufficient levels of purity for structural identification, e.g., with NMR. It is currently debated, if computational methods may compensate for the lack of purified references [165]. Depending on the acquisition method, different identification pipelines were developed by Eco-Metabolomics studies (Table 1).

With GC/MS, usually very robust capillary columns and precise ionization sources are used. This allows for rather predictable retention times and reliable spectral information and has enabled the set-up and use of large libraries such as NIST, MoNA or the Golm Metabolome Database (GMD) [5,166,167]. As a result, metabolite identification is more reliable when compared to LC/MS (see below). Identification of “unknown unknowns” is facilitated by using blind source separation and strategies that avoid hard chromatographic segmentation [168,169].

As LC/MS data are populated with different adducts, isotopes and in-source fragments, they are composed of many redundant features belonging to the same metabolite [170]. For peak grouping and annotation, many algorithms depend on the input of known *m/z* distances between common adducts, fragments and multiply charged ions. Despite the large number of bioinformatics tools and spectral libraries to annotate and identify metabolites (Table 3), the LC/MS-MS data processing pipeline is still very complex and time-consuming because it still involves extensive manual data inspection [6,11]. For metabolite identification, comparison to purified standard spectra in reference libraries is often necessary. The use of molecular networks, libraries such as the Global Natural Products Social (GNPS) Molecular Networking database and structural matching tools allow to compare the structural similarity of “unknown unknowns” with fragments of similar compounds that share a subset of the same sub-structures [171]. Furthermore, in this context, retention time prediction has been proposed as an additional, orthogonal property for the filtering of candidate compounds [172,173,174,175,176].

Many bioinformatics tools that perform in-silico prediction have been trained with known compounds mostly from model species such as *A. thaliana* or tomato [170,194]. Recent developments in computational annotation tools such as MetFrag and MetFamily enable to match measured spectra with reference spectra of compound classes. This allows for a more “fuzzy” match of features with similar spectra that enable more confident annotations of “unknown unknowns” [170,195]. In many cases, this “fuzzy” annotation may be sufficient for ecologists to explain certain biological observations, for example differences in herbivore resistance. More targeted analyses should follow to identify which of the compounds in a compound family, e.g., specific flavonoids, employ the causal agent for the effect. Machine learning approaches have additionally increased confidence of in-silico prediction of “unknown unknowns” with tools such as CFM-ID [179] and CSI:FingerID [180] (Table 3). Linking targeted spectral libraries with computational dereplication methods has been suggested to identify metabolites in non-model vs. model species [194,196,197].

### 5.4. Statistical Analyses

In Eco-Metabolomics, multi-factorial experiment designs and untargeted approaches leading to large data matrices with thousands of features necessitate appropriate statistical methods. Eco-Metabolomics studies listed in Table 1 used a plethora of different statistical methods. With targeted approaches, single features are usually compared between groups of samples with univariate statistical tests. The applicability of statistical tests depends on the number of predictors, the number of factor levels, the type of data (independent vs. dependent data) and the distribution of the data (e.g., normal vs. non-normal distribution, or homo- vs. heteroscedasticity). For Eco-Metabolomics studies, the non-parametric Kruskal–Wallis and Mann–Whitney U tests, as well as the parametric ANOVA have been successfully applied (Table 1). Nested designs are typically analysed with linear mixed effects models (lme), which allow to account for random factors and to find the correct error terms for the different hierarchical levels. If needed, post-hoc tests such as Tukey’s HSD can be applied to calculate p-values between different groups. However, when multiple metabolites or features are subjected to statistical analyses, false positives are a major concern (i.e., when the null hypothesis has been wrongly rejected). The Bonferroni correction (controlling the family wise error rate) or the Benjamini–Hochberg method (controlling the false discovery rate) were recommended by [198,199] for metabolomics in general. Several Eco-Metabolomics studies successfully applied the Holm–Sidak method and the Levene’s test for multi comparison correction [47,53,200,201] (Table 1).

To investigate multiple predictors, as well as to control for confounding parameters and replicate samples in a single approach, linear (mixed effect) models can also applied to multivariate data [4,47,202]. Beside tests for statistical significance, fold changes can be calculated for individual metabolites or features to indicate how strong their intensities differ between groups [198]. For metabolomics data, results from appropriate statistical tests can be combined with fold change analyses to judge which metabolites or features are interesting for further analyses [199].

With untargeted approaches, feature matrices sometimes have thousands of features and more complex statistical analyses are necessary due to the dimensionality of the data and the research questions. Principal Coordinate Analysis (PCoA) and (Non-metric) Multidimensional Scaling ((N)MDS) are two of the most frequent types of multivariate analysis used to compare metabolite profiles between samples and to select sets of metabolite candidates [203,204] (Table 1). The most interesting metabolites are often those with the largest differences between (several) groups. These are determined by performing post-hoc tests such as the non-parametric PERMANOVA (PERmutational Multivariate ANalysis Of VAriance) [198,205] (Table 1).

However, in Eco-Metabolomics there are often two or more data matrices, typically the feature table containing the biochemical information of the metabolite profiles and another matrix with many ecological parameters [141]. Here, ordination methods such as Redundancy Analysis (RDA), distance-based RDA (dbRDA) as well as Discriminant Analysis (DA) (esp. Linear DA), Orthogonal Partial Least Squares (OPLS), Hierarchical Clustering (HCA), classification and machine learning such as Random Forests (RF) and Support Vector Machines (SVM) are often applied to analyse two or more data tables conjointly. In this context, inclusion of meta-data from databases (see below) and data from, e.g., elemental analysers, can help to associate sets of metabolites with ecosystem functioning and to describe metabolomic traits [206] (Table 1; Figure 1c).

### 5.5. Bioinformatics Software Tools and Workflows

In many cases, bioinformatics software tools, data processing workflows and databases used in metabolomics were optimized for model species or were developed for clinical use cases. Thus, they are not directly applicable to Eco-Metabolomics studies. For non-bioinformaticians it is often hard to decide which software tools and which data sources are appropriate to the idiosyncratic Eco-Metabolomics experiments. Table 3 lists an overview of bioinformatics tools applicable to Eco-Metabolomics. Further information can be found in [132,157].

A major challenge in Eco-Metabolomics is that data repositories and libraries for both ecological and biochemical data are often fragmented. Some are not publicly accessible as they are owned by institutes or commercial parties. Furthermore, primary data are not always shared with the scientific community, are restricted to project members or are lost after a paper has been published [207]. Many biochemical databases such as PubChem or KEGG mainly contain chemical structures and information of model species. Databases such as KNApSAcK or NPASS can be used as a source of information regarding species-metabolite relations for non-model species [208,209]. Metabolic relationships and biochemical traits can be retrieved with databases such as MetaCyc [210], GMD [166] or BioCyc [211]. However, currently they do not allow for scaling up to processes at ecological scales.

Many Eco-Metabolomics studies rely on ecological data sources that are fragmented among countries or restricted to local floras (Table 1). The Plant Trait Database (TRY) agglomerates ecological traits of many different types of organisms globally and can also be used as a source in Eco-Metabolomics [212]. However, there remains the need for federated trait databases that aggregate the information from the many small local databases [213]. Even though there are no dedicated repositories for Eco-Metabolomics primary data, existing repositories such as MetaboLights, MetabolomicsWorkbench, MetabolomeExpress or GNPS are still rarely used to store raw profiles and to document meta-data [171,214,215,216] (Table 1).

Quality assurance and full reproducibility of the study are pivotal for good scientific practice [150]. In biochemistry, there are strict rules on analytical reproducibility of experiments. These have been part of good scientific practice and quality assurance for a long time [150]. For data processing and computational analyses, reproducibility is not always simple to achieve [207,217]. This is in part due to the complexity of biological and ecological systems [218], the diversity of technological platforms applied in metabolomics [136,219,220] and the large number of available bioinformatics tools. For example, [132] list more than 130 open bioinformatics tools, and many labs also use proprietary vendor software [221,222] (Table 3). To make it easier to discover related Eco-Metabolomics studies and to replicate experimental set-ups, it is recommended that data sets, meta-data and the corresponding bioinformatics data processing pipelines are shared with the scientific community [33,223]. Here, the FAIR guiding principles are a set of fundamental rules that contribute to good data management and stewardship (long-term care) [207]. The FAIR acronym stands for Findability, Accessibility, Interoperability and Reusability of data (Table 4) and following these rules can make Eco-Metabolomics data sets available to a broader scientific audience [207].

In the last years, bioinformatics workflow platforms have been set up to cover all the required steps of the data processing pipeline, beginning with data download from a public repository, data quality control [224] and the various biostatistical analyses (see above). It is vital to reproduce the data processing pipeline to allow the scientific community to get reliable insight at any level of the study [225]. Currently, scientists often struggle with repeating certain steps due to the technical complexity of the software used. In recent years, the Galaxy workflow platform has become increasingly popular with many “omics” technologies [226]. For Eco-Metabolomics, several existing dedicated metabolomics workflow platforms can be used, such as the Galaxy workflow systems, Workflow4Metabolomics [192] and Galaxy-M [181] as well as the KNIME workflow system which already has some mass spectrometry related OpenMS modules integrated [227,228]. However, these workflow platforms need to be improved to also contain dedicated Eco-Metabolomics modules to facilitate data processing for non-bioinformaticians [33].

## 6. Possible Limitations in Eco-Metabolomics

As normally metabolites in organisms are in a known steady-state level (homeostasis), deviations can be measured using metabolomics techniques. In systems biology these deviations are the basis for modelling and allow to scale up from spatiotemporally fine to coarser scales [106]. However, comparing metabolite profiles from samples collected in the morning with profiles from samples taken in the evening from identical plant individuals may result in largely different profiles as shown, e.g., for *Arabidopsis*, *Silene* and CAM plants [21,63,144,145,229]. Thus, a metabolite profile is always static and is merely a snapshot of biochemistry at a fixed point in time. Furthermore, as the biotic environment can mitigate the effects of, for example, climate change, it is important in Eco-Metabolomics to not only measure profiles of individuals of target species, but also to consider other plants in the community and the properties of the surrounding ecosystem [96]. As endophytic microorganisms can colonise internal tissues of host plants, they can influence metabolite profiles of plants and may even contribute exogenous metabolites [230]. Endophytes can form various relationships with their host plants, which can be detrimental (e.g., pathogenic fungi), but also beneficial to both partners (e.g., symbiotic, mutualistic or commensalistic) [230,231]. This sometimes makes it challenging to draw conclusions from spatiotemporally fine to coarser scales, which are nonetheless very important to estimate impacts on ecosystem services [157].

While the sensitivity of analytical platforms can be a “blessing” in bottom-up approaches in biochemistry, and has enabled many detailed insights into processes within organisms that would not have been possible to be detected otherwise, it could also be a “curse” with top-down approaches [94]. In ecology, the sensitivity of metabolite profiles may include undesired short-term fluctuations (e.g., unsteady weather conditions) and, thus often necessitates to take many metabolite profiles and to measure additional non-biochemical traits of plants and environmental conditions to rule out side-effects or to correct for shifts and fluctuations [94]. In ecology, there are many statistical methods that were designed explicitly for dealing with large variability [141]. However, biostatistics cannot compensate for poor experimental designs. It is also important to consider that biochemical traits may not be involved in every type of ecological interactions [94]. For these reasons it is of uttermost importance for top-down Eco-Metabolomics studies, that a sharp research question or hypothesis is defined even before the samples are taken.

No single analytical method can cover all metabolites at once. Indeed, the metabolite coverage highly depends on the extraction method and type of instrumentation used. It is beyond the scope of this review to list the strengths and weaknesses of the various types of acquisition methods. We refer the interested reader to other review papers, e.g., [5,7,8,14,94,170] (refer also to Table 2).

## 7. Future Directions in Eco-Metabolomics

We argue that Eco-Metabolomics provides novel approaches to answer fundamental ecological questions. For example, many processes in ecology are driven by interactions, such as those between soil microbes and plants, which are invisible to the human eye [232]. In ecology, organismal interactions, or linkages, can be visualized with, e.g., Structural Equation Modelling (SEM), which can model relationships between multivariate data with cause–effect equations at different scales [233,234,235,236,237,238]. As metabolites are mediators in these processes, they have the potential to be used for SEM [239,240,241,242]. As SEM is similar to approaches taken in systems biology, where processes in cells are modelled [106], we suggest that SEM are used as a tool in Eco-Metabolomics in the future. This will allow measuring the number and strengths of ecological linkages and, thus, visualising the various types of biochemical interactions organisms realize in ecosystems. Furthermore, latent variables in SEMs may be used to construct “eco-chemical” traits that explain part of the functioning of ecosystems.

We have discussed that with the top-down approaches commonly used in Eco-Metabolomics it is often not feasible to identify singular metabolites. Rather identifying sets of metabolite features, annotating compound classes and linking them to ecological function are some of the main objectives. The answers obtained by these types of studies can be used to derive and construct new questions and hypotheses that are of vital interest for biochemistry. Thus, after identifying basic ecological relationships, the results can be used for “zooming in” and performing bottom-up approaches that give new detailed insights into ecological processes mediated by metabolites. However, it should be considered that metabolomics in principle is a hypothesis generating approach. If the goal of the study is to pinpoint specific metabolites responsible for ecological interactions, top-down studies must be followed by manipulative experiments.

In this review, we have discussed two approaches to answer fundamental ecological questions. Both bottom-up and top-down approaches have unique strengths and challenges and can contribute greatly to science. We conclude that experimental designs will likely get more complex in the future and that more factors will be incorporated in the studies. Furthermore, to increase the metabolite coverage, a combination of several extraction methods and analytical platforms may even be used in the future. There has also been a shift towards the use of non-model species with both types of approaches. The challenges we identified in this review are not limited to the plant domain. In fact, other domains such as metabolomics analyses of wild animals have similar challenges, e.g., dealing with “unknown unknowns” and with non-model species.

We ascertained that the term “Eco-Metabolomics” is not used widely by the scientific community. Publishing Eco-Metabolomics primary data in repositories once studies have been published will greatly contribute to make Eco-Metabolomics available to a broader scientific audience and will allow metadata studies that re-analyse results from the various Eco-Metabolomics studies in the future. However, there is still the need for a better integration between spatiotemporal scales, a closer collaboration between researchers to improve databases for both non-model species and spectral information and, thus, also among scientists around the world.

## Figures and Tables

**Table 1:** Overview of selected research studies in the field of Eco-Metabolomics in the last decade. The table was ordered by the columns “Approach”, “Interaction level” and “Non-model species”. Bottom-up in the column “Approach” defines an approach typically taken by biochemists who infer from spatiotemporally fine scales such as from molecular and physiological scales within plants to spatiotemporally coarser scales. Top-down defines an approach typically taken by ecologists who infer from spatiotemporally coarse scales such as community and population scales to intrinsic scales within plants. “Interaction level” refers to the type of ecological or biological interaction which has been analysed in the study. The column “Non-model species” refers to whether a model species such as *A. thaliana*, rice or tomato was used in the study. The column “Experimental methodology” lists the type of environment in which the study was performed. “Metabolomics acquisition method” refers to the type of metabolomics technology that was used in the study.

Reference	Approach	Interaction Level	Non-Model Species?	Plant Species Studied	Experimental Methodology	Metabolomics Acquisition Method	Statistical Methods	Bioinformatics Tools Used	Compounds Identified	Key Results
[35]	top-down	plant–diversity	yes	*Bellis perennis Knautia arvensis Lotus corniculatus Medicago x varia Leontodon autumnalis*	field	GC/MS FT-ICR-MS	GLM, PCA, ANOVA, HCA, Kruskal–Wallis test		yes	Negative effects of resource competition with small-statured species, modified metabolite profiles in response to altered resource availability with tall species
[36]	top-down	plant–diversity	yes	*Festuca pratensis Poa pratensis Plantago lanceolata Prunella vulgaris Crepis biennis Galium mollugo Onobrychis viciifolia Trifolium repens*	semi-field plots	FTIR	LDA, Canonical variate analysis, NMDS, HCA		classes	Metabolic profiles of species can be differentiated according to the diversity level they grew in
[37]	top-down	plant–environment	yes	*Lepidium latifolium*	field	HPLC	ANOVA, Tukey HSD	SPSS	yes	The species (also described as “sleeper weed“) has biochemical plasticity in response to different environments
[38]	top-down	plant–environment	yes	*Carex caryophyllea*	growth chamber	LC/MS	PCA, DCA, Pearson correlation	SIMCA-P, PC-ORD	no	Interaction of genetic diversity and resulting metabolite plasticity with regard to soil type and environment
[39]	top-down	plant–environment	yes	*Poa annua Poa cookii Poa kerguelensis Ranunculus biternatus Ranunculus pseudotrullifolius Ranunculus moseleyi Pringlea antiscorbutica Acaena magellanica Taraxacum erythrospermum*	field	HPLC	discriminant analysis, ANOVA	StatSoft	yes	Differences in amine composition can be linked to environment
[40]	top-down	plant–environment	yes	*Artemisia biennis Artemisia vulgaris Bidens frondosa Bidens tripartita Senecio inaequidens Senecio vulgaris Senecio jacobaea Solidago gigantea Solidago virgaurea Tanacetum parthenium Tanacetum vulgare Tragopogon dubius Tragopogon pratensis*	greenhouse	LC/MS	ANOVA, Spearman correlation	Metalign, R	no	Exotic species have more and also more unique metabolites when compared to native congeners, herbivore performance was lower with exotics
[41]	top-down	plant–environment	yes	*Brachythecium rutabulum Calliergonella cuspidata Fissidens taxifolius Grimmia pulvinata Hypnum cupressiforme Marchantia polymorpha Plagiomnium undulatum Polytrichum strictum Rhytidiadelphus squarrosus*	field	LC/MS	dbRDA, HCA, ANOVA, Tukey HSD, Pearson correlation, Mantel test	R, CompassXPort, CompassIsotopePattern, CompassDataAnalysis, ISAcreator, Docker, Galaxy	no	Patterns in metabolite profiles of bryophytes are connected to phylogenetic history, seasonal changes, ecological characteristics and life strategies
[42]	top-down	plant–environment	yes	*Myriophyllum spicatum*	field	GC/MS	*t*-test, PCA	R, XCMS	no	Metabolite profiles are related to ontogenetic development, habitat and nutrient status of lake
[43]	top-down	plant–environment	yes	*Quercus acutissima Schima superba Sapindus saponaria*	field	LC/MS-MS HPLC	ANOVA, F-test, NMDS, RDA	R	yes	Litter diversity effects on the decomposition of leaf litter tannin and polyphenols of three tree species
[44]	top-down	plant–environment	yes	*Erica multiflora*	field	CHNS-O elemental analyser NMR	MANOVA, PERMANOVA, PCA, DA	TOPSPIN, PRIMER, Statistica	yes	Stoichiometrical evidence for the growth-rate hypothesis
[45]	top-down	plant–environment	yes	*Quercus ilex*	field	LC/MS NMR CEM	PERMANOVA, ANOVA, PCA, PLS-DA, GLM	R, TOPSPIN, AMIX, Statistica	classes	Drought shifts metabolism as plants adapt metabolism and folivory to prevent water loss
[46]	top-down	plant–herbivore	yes	*Inga marginata Inga acreana Inga auristellae Inga tenuistipula Inga umbellifera Inga laurina*	field	LC/MS	PCA, HCA, PLS-DA, Venn, ANOVA, Kruskal–Wallis test	R, MetaboAnalyst	no	Metabolomics and advances in bioinformatics allow For comprehensive examination of shifts in foliar chemical defenses of trees depending on leaf development stage
[47]	top-down	plant–herbivore	yes	*Bunias orientalis*	growth chamber	LC/MS	linear (mixed effect) model, ANOVA, NMDS, Mantel test, Spearman rank correlation, Shannon diversity, Holm-Sidak, Levene's test	R	yes (glucosinolates)	Genetic distances of 16 *Bunias orientalis* populations correlated with metabolite fingerprints; invasion success is facilitated by high metabolite variation and diversity within populations which play a role with reducing herbivory to the herbovore *Mamestra brassicae*
[48]	top-down	plant–herbivore	yes	*Inga heterophylla Inga capitata*	field	GC/MS LC/MS	PCA, PLS-DA	R, Metaboanalyst	yes	interactions with natural enemies play a significant role in phenotypic divergence and potentially in diversification and coexistence of two tropical sister species; defensive traits are evolutionary labile
[49]	top-down	plant–herbivore	yes	*Bunias orientalis*	glasshouse	LC/MS	linear mixed model, REML, Tukey-Kramer test, PCA, ANOVA	SAS, R	classes (glucosinolates)	Native populations are better defended against herbivory than non-native populations
[50]	top-down	plant–herbivore	yes	*37 Inga species*	field	LC/MS	HCA, PCA, Bayesian	R, MrBayes, MacClade	classes	Species of Inga trees that co-occur at local and regional spatial scales are less similar in terms of their metabolomes than by chance, suggesting that interactions with shared herbivores and pathogens (whose host ranges are determined by the trees’ metabolomes) select for chemically diverse plant assemblages, and hence facilitate ecological coexistence in the tree community (in this case among congeneric trees)
[51]	top-down	plant–herbivore	yes	*Barbarea vulgaris subsp. arcuata*	growth chamber	LC/MS	*t*-test, correlation, regression, HCA, PCA	MetAlign, Java, SAS, R	yes + classes	Metabolite profiles differentiated plants susceptible to the herbivore Phyllotreta nemorum, the known compounds hederagenin cellobioside and oleanolic acid cellobioside, as well as two other saponins were correlated with plant resistance
[52]	top-down	plant–herbivore	yes	*Daucus carota*	growth chamber	NMR	Pearson correlation, ANOVA, PCA, PLS-DA, OPLS-DA	TOPSPIN, SIMCA-P	yes	Wild carrots are more resistant to herbivores than cultivated species + identification of compounds that are important for interaction
[53,54,55]	top-down	plant–herbivore	yes	*Pinus sylvestris ssp. nevadensis Pinus sylvestris ssp. iberica Pinus pinaster Pinus nigra Pinus nevadensis*	field	LC/MS	Shapiro–Wilk, ANOVA, Levene's test, PERMANOVA, Tukey's HSD, PCA, Euclidean distance, PERMANOVA, PLS-DA, HCA	R, MZmine	no	The metabolomes of the tested Pinus species were more dissimilar to folivory in summer than in winter possibly due to drought conditions
[56]	top-down	plant–herbivore	yes	*46 tree species from four genus-level clades, including Eugenia (4 species), Inga (14 species), Ocotea (including Nectandra; 8 species) and Psychotria (including Palicourea; 20 species)*	field	LC/MS LC/MS-MS	Chemical structural compositional similarity, Bray-Curtis similarity, Permutation test	GNPS, R	yes (in Supporting Information)	Interspecific differences, including those among congeneric species of trees, were much larger than within species and chemical structural similarity of ontogeny, light environment and season. Variation between metabolite profiles permits niche segregation among congeneric tree species based on chemical defences.
[57]	top-down	plant–herbivore	no	*Zea mays ssp. mays Zea mays ssp. parviglumis*	glasshouse	LC/MS	linear mixed model, ANOVA, PLS, MANOVA		yes (BXDs)	Domesticated maize plants have weakened chemical defences against several herbivores when compared to teosinte, the wild maize ancestor
[58]	top-down	plant–herbivore	no	*Nicotiana attenuata*	greenhouse	LC/MS LC/MS-MS	Coexpression networks, PCA	R, Cytoscape	yes	Metabolic branch-specific variations in natural accessions identified by fragmentation analysis, discovery and annotation of ecologically interesting compounds
[59]	top-down	plant–pathogen	yes	*Piper santi-felicis Piper multiplinervium Piper cenocladum Piper reticulatum Piper holdrigeanum Piper auritum Piper xanthostachym Piper peltatum Piper melanocladum*	field	NMR	Diversity indices, a priori path models (PROC CALIS), upfield and downfield diversity	MestReNova, SAS	classes	Elevated phytochemical diversity in 9 *Piper* species has positive effects on the diversity of herbivores and reduces overall herbivore damage. Metabolite profiles provide mechanistic evidence for the predominance of specialized insect herbivores on *Piper*
[60]	top-down	plant–plant	yes	*Pinus halepensis Quercus pubescens*	field	GC/MS	ANOVA, Tukey test, *t*-test, PCA, SIMPER, Mann–Whitney test	R, PRIMER-E, GraphPad	no	Plants modulate their metabolism (trade-off of allelopathy and growth) according to level of competition
[61]	top-down	plant–plant	yes	*Plantago lanceolata*	greenhouse	HPLC	linear mixed model, Tukey HSD	R	no	Phenotypic plasticity in response to environmental variation rather than genetic differentiation as a response to plant diversity
[62]	top-down	plant–plant	yes	*Karenia brevis Asterionellopsis glacialis Thalassiosira pseudonana*	cultures	LC/MS NMR	PCA, PLS-DA	Matlab, PLS_Toolbox, SEQUEST, NMRLab, MassLynx	yes	Allelochemicals target multiple pathways in competitors, affecting primary production and nutrient cycling in ecosystems
[63]	top-down	plant–pollinator	yes	*Silene otites*	field semi-field plots	GC/MS	non-parametric ANOVA, Tukey-Kramer post hoc test	Saturn Software, MassFinder, Statistica	yes	Diel variation in floral volatile composition, emission patterns correspond to olfactory ability and activity times of insect pollinators
[64]	top-down	plant–soil	yes	*Holcus lanatus Alopecurus pratensis*	field	LC/MS NMR	PERMANOVA, PCA, PLS-DA, ANOVA, Kolmogorov-Smirnov test	MZMINE, TOPSPIN, AMIX, Statistica, R	yes	Different responses of species to environmental stresses, responses opposite in shoots and roots
[65]	top-down	plant–soil	yes	*Sambucus nigra*	field	LC/MS	PERMANOVA, PCA, PLS-DA, ANOVA, Kolmogorov-Smirnov test	MZMINE, Statistica, R	yes	Microbial communities in the phyllosphere have impact on metabolome of plants
[66]	bottom-up	plant–environment	yes	*Pseudotsuga menziesii*	growth chamber	GC/MS	*t*-test	SigmaPlot, Excel	yes	Provenance-specific reactions to environmental stress as outlined with identifying specific compounds
[67]	bottom-up	plant–environment	yes	*Ostreococcus tauri*	cultures	GC/MS	none	Xcalibur, MET-IDEA, Excel, AMDIS, MS Search	yes	Metabolomes show diurnal fluctuations + identification of formerly unknown metabolites
[68]	bottom-up	plant–environment	yes	*Echium plantagineum Echium vulgare*	glasshouse	LC/MS	Logistic regression	MassHunter, Statistix, Excel	yes	Role of shikonins in relation to plant phenological stage
[69]	bottom-up	plant–environment	yes	*Cistus ladanifer*	field	HPLC	HCA, ANOVA	-	yes	Intra-population variation in the metabolomes with regard to environment
[70]	bottom-up	plant–environment	no	*Synechococcus elongatus*	cultures	LC/MS LC/MS-MS	Pearson correlation, Spearman correlation, NMDS, ANOSIM	XCalibur, Excel, R, Metlin, MetFrag, KEGG, MetaboLights	yes	Exuded metabolites to the environment have ecological relevance on e.g., microbes
[71]	bottom-up	plant–environment	no	*Zea mays*	greenhouse	NMR	ANOVA, PCA, HCA, linear regression	SIMCA-P+, SPSS	yes	Plastic responses of different maize lines to temperature conditions
[72]	bottom-up	plant–environment	no	*Solanum lycopersicum*	greenhouse	LC/MS	OPLS-DA, ANOVA	SIMCA	yes	Metabolome of tomato changes with different salinity levels, carotenoid accumulation with higher salinity was observed
[73,74,75]	bottom-up	plant–fungusplant–herbivore	yes	*Plantago major Plantago lanceolata Veronica chamaedrys Medicago truncatula Poa annua*	growth chamber climate chamber	GC/MS LC/MS LC-FL elemental analyser	cluster heatmap average linkage, HCA, Pearson correlation, GLM, Mann–Whitney U test, Kruskal–Wallis test, Dunn test, volcano plot, Chi2 test, Venn-Euler diagram	MassHunter, Xcalibur, XCMS, R, Excel, GLM, Cluster, JavaTreeView, MATLAB, KEGG	yes	There is a core-Metabolome across species and a phytometabolome which is species-specific as a response to arbuscular mycorrhizal fungus. Foliar metabolome modifications are determined by the developmental stage of arbuscular mycorrhiza with changes becoming more pronounced over time and being only partly phosphate-mediated. Specific effects of jasmonic acid and salicylic acid on metabolite pattern in leaf tissue and phloem exudates.
[76]	bottom-up	plant–herbivore	yes	*Solanum dulcamara*	greenhouse	LC/MS	Friedman ANOVA, Wilcoxon signed-rank test, Pearson's correlation test and heatmap	MetaboAnalyst 3.0	yes	Variation in steroidal glycoalkaloids (GAs) correlated with slug preference; accessions with high GA levels were consistently less damaged by slugs. One, strongly preferred, accession with particularly low GA levels contained high levels of structurally related steroidal compounds. These were conjugated with uronic acid instead of the glycoside moieties common for *Solanum* GAs.
[77]	bottom-up	plant–herbivore	yes	*Plantago lanceolata*	growth chamber	LC/MS GC/MS	GLM, Kruskal–Wallis test, PCA, Mann–Whitney U test, volcano plot, Chi2 test, Venn-Euler diagram	MassHunter, Xcalibur, XCMS, R, Excel, MATLAB, VennMaster	yes	Metabolic fingerprints were considerably affected especially by generalist and phytohormone treatments, but less by mechanical damage and specialist herbivory. Responses to generalists partly overlapped with the changes due to jasmonic acid, but many additional peaks were up-regulated. Many features were co-induced by jasmonic and salicylic acid.
[78]	bottom-up	plant–herbivore	yes	*Brassica oleracea*	greenhouse	LC/MS LC/MS-MS	PCA, PLS-DA	Metaboanalyst 3.0	yes	Results showed that Xcc infection causes dynamic changes in the metabolome of *B. oleracea*. Repression pattern of the metabolites implicated in the response follows complex dynamics during infection progression indicating a complex temporal response. Specific metabolic pathways such as alkaloids, coumarins or sphingolipids are identified as candidates in the infection response
[79]	bottom-up	plant–herbivore	no	*Oryza sativa*	growth chamber	LC/MS LC/MS-MS	ANOVA, LSD, PCA, *t*-test	MetaboAnalyst, Excel	yes	Identification of formerly unknown compounds in rice in response to herbivory
[80]	bottom-up	plant–herbivore	no	*Brassica oleracea*	climate chamber	HPLC CHN elemental analyser	ANOVA, LSD test, *t*-test	PASWStatistics	yes	Responses of herbivores and their interactions with host plants are depending on drought stress
[81]	bottom-up	plant–herbivore	no	*Nicotiana attenuata*	climate chamber	LC/MS	PCA, Shapiro–Wilk test, *t*-test, linear mixed model, REML	MetaboAnalyst, R	yes	Damage-induced defence may undergo circadian fluctuation
[82]	bottom-up	plant–herbivore	no	*Arabidopsis thaliana*	growth chamber	GC/MS LC/MS	Kruskal–Wallis, Tukey HSD, Mann–Whitney U test, *t*-test, Spearman correlation, GLM, PCA, OPLS-DA, ANOVA	XCalibur, Agilent MassHunter, SIMCA, R	yes	Systemic plant responses to nematode and aphid interferences
[83]	bottom-up	plant–herbivore	no	*Arabidopsis thaliana*	growth chamber	GC/MS elemental analyser	PCA, PLS-DA, two-way ANOVA	XCalibur, R	yes	Effects of aphid shoot feeding on root metabolite profiles depend on fertilization, leading to contrasting effects on nematodes
[84]	bottom-up	plant–herbivore	no	*Nicotiana tabacum*	growth chamber	NMR GC/MS	PCA, OPLS-DA	SIMCA-P+	yes	Conclusions for plant defence mechanisms following infection of leafy gall
[85]	bottom-up	plant–plant	yes	*Populus alba Populus tremula*	field	LC/MS	PCA, ANOVA, LSD test, Mann–Whitney U test, Mantel test	Markerlynx XS, SPSS	yes	Linking chemical traits to genotypic evolution
[86]	bottom-up	plant–plant	yes	*Chaetoceros socialis*	cultures	LC/MS	Mann–Whitney U test, Spearman correlation, PCA	Statistica, MarkerLynx XS, Excel	no	linking metabolite profiles to phenotypic differences, phylogeny and temperature regimes
[87]	bottom-up	plant–plant	yes	*Heracleum mantegazzianum*	greenhouse	LC/MS	linear mixed models, variance component analysis, OPLS, ANOVA,	R, MetAlign, SIMCA-P	yes	Intraspecific variability is important with allelopathy + identification of some compounds

**Table 2:** List of related review papers that deal with specific questions related to Eco-Metabolomics. The table was ordered by means of the columns “Approach”, “Spatiotemporal scales covered” and “Interaction level”. Bottom-up in the column “Approach” defines an approach typically taken by biochemists who infer from spatiotemporally fine scales such as from molecular and physiological scales within plants to spatiotemporally coarser scales. Top-down defines an approach typically taken by ecologists who infer from spatiotemporally coarse scales such as community and population scales to intrinsic scales within plants. The column “Spatiotemporal scales covered” list the scale levels which have been covered. “Interaction level” refers to the type of ecological or biological interaction which have been covered in the review paper. “Metabolomics acquisition methods” refers to the type of metabolomics technology that have been described in the paper. The column “Contribution of metabolomics” list the value that metabolomics contributes to research.

Reference	Approach	Spatiotemporal Scales Covered	Interaction Level	Metabolomics Acquisition Methods	Contribution of Metabolomics
[88]	top-down	Community Population Individual	plant–herbivore plant–pathogen	-	Multitrophic interactions within a web of species interactions are mediated by phytochemicals that can be determined with metabolomics. These phytochemicals influence and trigger immune responses in both plants and herbivores/pathogens.
[89]	top-down	Community Population Individual	plant–herbivore plant–pathogen plant–plant	NMR LC/MS, LC/MS-MS	Metabolomics can reveal cryptic biochemical traits that mediate interactions of plants with other organisms; emphasis on species coexistence, lineage diversification and character evolution and potential of metabolomics
[90]	top-down	Community Population Individual Physiology Molecular	plant–plant plant–community	GC/MS	Central role of metabolomic traits that can describe species coexistence chemically, Metabolomics can be used to detect the genetic identity of neighbours if they have common history of coexistence
[91]	top-down	Landscape Community Population	plant–environment plant–community plant–plant	-	Metabolomics and chemical/ecophysiological interactions can be used to describe plant traits and phenotypic plasticity
[92]	top-down	Landscape Community Population Individual Physiology Molecular	plant–environment	LC/MS GC/MS NMR HPLC	Climate change acts on various scales on plants and affects their phenotypic plasticity, genotypic evolution, migration and local extinction of populations and result in biogeochemical and biophysical feedbacks: The potential of metabolomics are highlighted
[93]	bottom-up top-down	Community Population Individual Physiology	rhizosphere community plant–plant plant–herbivore plant–pathogen plant–community	GC/MS LC/MS NMR	Metabolomics can help to understand interactions of plant roots and organisms in the rhizosphere
[94]	top-down bottom-up	Community Population Individual	plant–plant plant–herbivore plant–community	FTIR NMR UV	Metabolomics can provide new insight into ecological processes such as interactions of plant with pollution, biotic and environmental stress
[95]	top-down bottom-up	Community Population Individual Physiology Molecular	plant–environment	GC/MS LC/MS NMR HPLC Fluorescence microimaging	Metabolomic approaches (untarged + targeted) can provide powerful insights at various scales
[96]	top-down bottom-up	Landscape Community Population	plant–environment	GC/MS LC/MS NMR	Metabolite profiles of model species can be used to determine ability of plant to recover from stress but also for stress-buffering capacities of ecosystems
[10]	top-down bottom-up	Population Individual Physiology	plant–environment plant–herbivory	LC/MS GC/MS FT-ICR NMR	Ecophysiological responses of plants to temperature, water, nutrients, light/circadian rhythm, atmospheric gases, seasonality; differentiation of aquatic and terrestrial organisms; emphasis on field studies and variation; biotic interactions
[97]	bottom-up	Community Population Individual Physiology	plant–plant	-	With plant–plant interactions, especially competition, sensing of compounds through light-quality signals, nutrient levels, soluble root exudates and volatile organic compounds emitted by neighbouring plants both above- and below-ground is vital
[98]	bottom-up	Community Population Individual Physiology Molecular	rhizosphere community	-	Metabolic pathways of microbes in the rhizosphere can be modelled with meta-genomic sequencing data and systems biology approaches. Systems biology approaches enable scale-independent thinking.
[7]	bottom-up	Individual Physiology	plant–environment	NMR LC/MS, LC/MS-MS GC/MS FT-ICR DIMS	Potential and challenges of environmental metabolomics with emphasis on analytical techniques
[99]	bottom-up	Individual Physiology	plant–fungus	GC/MS LC/MS	Mycorrhiza-mediated changes in foliar metabolome are highly species-specific and cover many different compound classes; changes can confer protection against abiotic stresses and have consequences on numerous biotic interactions
[100]	bottom-up	Individual Physiology	plant–herbivore	GC/MS LC/MS	Role of system-wide untargeted metabolomics analysis for plant–herbivore interactions with emphasis on analytical and statistical methods
[101]	bottom-up	Individual Physiology	plant–pathogen	NMR	Application of NMR in metabolomics and its role in detecting host plant resistance to pathogens
[102]	bottom-up	Individual Physiology Molecular	plant–environment plant–herbivore plant–pathogen	GC/MS LC/MS, LC/MS-MS NMR	Metabolomics can provide detailed insights into ecological interaction processes; Targeted and comparative metabolomics can reveal new and important compounds involved with these interactions; general analytical and statistical approaches are discussed
[103]	bottom-up	Individual Physiology Molecular	plant–environment plant–plant plant–herbivory plant–pathogen systems biology	GC/MS LC/MS NMR	General contribution of metabolomics from a systems biological view point
[104]	bottom-up	Individual Physiology Molecular	plant–pathogen plant–mutualist plant–microbes	GC/MS LC/MS FIE-MS FT-ICR-MS	Metabolomics can provide improved spatial and temporal separation of biotrophic interaction processes between plants and pathogenic + mutualistic fungi
[105]	bottom-up	Individual Physiology Molecular	plant–environment	GC/MS LC/MS NMR LIF	Ecophysiological responses of plants to drought, cold stress, salinity + integration of several Omics
[106]	bottom-up	Landscape Community Population Individual Physiology Molecular	plant–environment systems biology	GC-MS LC/MS UPLC Proteomics	Practical applications necessitate in-depth understanding of the physiology of single plant species; Metabolomics is one key technology to translate this knowledge to complex ecosystems; Correlation networks are one way to determine multi-scale interactions
[107]	bottom-up	Landscape Population Individual Physiology	plant–environment	-	Metabolomics can identify biomarkers and contaminants involved with environmental pollution; Metabolomics can be used to develop policies and management for sustainable environments; The concept of scaling and levels of biological organisation are discussed
[108]	bottom-up	Population Individual Physiology	plant–environment	LC/MS GC/MS NMR	General overview on experimental design, extraction methods, analytical instrumentation and statistical methods used in environmental metabolomics and pipeline how to detect biomarkers
[109]	bottom-up	Population Individual Physiology Molecular	plant–herbivore	LC/MS GC/MS NMR FTIR	Metabolomics is a research domain linking genotypes to phenotypes, describing metabolites that are important in plant herbivore interactions

**Table 3:** List of bioinformatics tools applicable to use in Eco-Metabolomics.

Bioinformatics Tool	Reference	Metabolomics Acquisition Methods Covered	Main Functionality
AMDIS	[177]	GC/MS	Spectrum deconvolution, identification
BATMAN	[178]	NMR	Identification and quantification of metabolites in deconvoluted NMR data
CAMERA	[162]	GC/MS, LC/MS	Feature annotation, feature alignment, RT correction, isotope cluster validation
CFM-ID	[179]	LC/MS-MS	Identification, Spectrum prediction
CSI:FingerID	[180]	LC/MS-MS	Identification
Galaxy-M	[181]	LC/MS	Workflow system for metabolomics data analysis
GNPS	[171]	LC/MS-MS	Retrieval of online dereplicated and crowdsourced MS/MS spectra
iMet	[182]	LC/MS-MS	Identification
MetaboAnalyst	[183]	NMR, LC/MS, GC/MS	User interface for the processing and analysis of metabolomics data
MetFamily	[184]	GC/MS, LC/MS	Clustering of MS features to metabolite families
MetFrag	[185]	LC/MS-MS	Identification of MS features by their MS-MS spectra
MS2LDA	[186]	LC/MS-MS	Decomposition of MS/MS spectra to co-occurring fragments/neutral losses
MS-Dial	[187]	LC/MS-MS, GC-MS	Processing, deconvolution and analysis of MS data
mzMatch	[188]	GC/MS, LC/MS	Tool chain for the processing of metabolomics data
MZmine 2	[189]	LC/MS	Framework for the processing and analysis of MS data
OpenMS	[156]	GC/MS, LC/MS	Feature extraction and data analysis
NMRProcFlow	[190]	NMR	Processing and visualization of 1D NMR data
SIRIUS	[191]	LC/MS	Annotation of sum formulas using MS/MS spectra and isotope patterns
Workflow4Metabolomics	[192]	NMR, LC/MS, GC/MS	Automatic processing, annotation and analysis of metabolomics data
XCMS	[155]	GC/MS, LC/MS	Feature extraction
XCMS Online	[193]	GC/MS, LC/MS	User interface for processing and analysis of metabolomics data

**Table 4:** FAIR criteria for the reuse of data as described in [207].

Criteria	Summary of Execution
**Findability**	(meta)data are assigned globally unique and persistent identifiers which are registered and indexed in searchable resources
**Accessibility**	(meta)data are retrievable by their identifier with an open and free protocol, metadata are still accessible even when data is no longer available
**Interoperability**	(meta)data use formal, accessible, shared and broadly applicable language and have vocabularies that follow FAIR principles and include qualified references to other (meta)data
**Reusability**	(meta)data are associated with accurate and relevant attributes, with detailed provenance, with an accessible license and meet domain-relevant community-standards

## Supplementary Materials

Supplementary File 1
